# Effectiveness of Recent Physiotherapy Techniques Along With Conventional Physiotherapy Techniques in a Patient With Knee Osteoarthritis: A Case Report

**DOI:** 10.7759/cureus.54872

**Published:** 2024-02-25

**Authors:** Kamya J Somaiya, Subrat Samal, Manali A Boob

**Affiliations:** 1 Musculoskeletal Physiotherapy, Ravi Nair Physiotherapy College, Datta Meghe Institute of Higher Education and Research, Wardha, IND

**Keywords:** low-intensity plyometrics, kinesio taping, quality of life, mulligan mobilisation, knee osteoarthritis

## Abstract

Osteoarthritis (OA), the most common joint disease, lowers quality of life, restricts social activity participation, and results in incapacity. Osteoarthritis is characterised by changes in subchondral bone, meniscus degeneration, cartilage loss, and synovial inflammation. Physiotherapy plays a vital role in maintaining the stability of this disease. Various treatment approaches have been shown in numerous studies to be successful in improving the condition of individuals with osteoarthritis in the knee. We are presenting a case of a 47-year-old woman who had bilateral osteoarthritis in her knees. We created a six-week treatment plan for this patient that incorporates a number of advanced therapy techniques, including Mulligan mobilisation, Kinesio taping, and plyometric exercise sessions. We created a thorough rehabilitation programme for our patient, who had osteoarthritis in her knee, and it worked incredibly well. We assessed the efficacy of our outcome measures using a variety of outcomes, including the Western Ontario and McMaster Universities Osteoarthritis Index (WOMAC), Knee Injury and Osteoarthritis Outcome Score (KOOS), visual analogue scale (VAS), range of motion (ROM), and manual muscle testing (MMT). It was found to be more beneficial to provide modern physiotherapeutic approaches in addition to a traditional physiotherapy course for improving the overall health and quality of life of the patient.

## Introduction

The most prevalent joint condition, osteoarthritis (OA), reduces quality of life, limits participation in social activities, and causes disability [1]. Primary and secondary osteoarthritis are the two categories of the condition. Articular degeneration in primary osteoarthritis has no discernible underlying cause. Either aberrant articular cartilage, as in rheumatoid arthritis (RA), or an aberrant concentration of force across the joint, as in post-traumatic reasons, can result in secondary osteoarthritis. Usually, osteoarthritis progresses over time and can eventually cause disability [2]. It was discovered that 28.7% of people had knee OA overall. The findings indicated that age, sedentary work, obesity, and female gender (prevalence of 31.6%) were linked factors. This study has shown that a significant portion of the population has borderline OA; as a result, awareness campaigns that aim to eliminate modifiable risk factors are the primary means of maintaining senior patients' comfort and mobility [3]. Several types of physiotherapy are used as a non-invasive approach for treating osteoarthritis. Numerous studies have demonstrated how different therapy modalities can effectively improve the condition of people with knee osteoarthritis. Realigning the patella has been found to be highly successful using knee taping. Although therapist contact may enhance benefits, all delivery modes - individual, group, and home exercise - are beneficial. To optimise results over the long run, the focus must be placed on enhancing exercise adherence [4]. Numerous studies have been conducted to demonstrate their efficacy in this particular disease. Physiotherapy is a critical component of keeping this condition stable. We are showcasing the case of a 47-year-old female with osteoarthritis in the bilateral knee. We created a thorough rehabilitation programme for our patient, who had osteoarthritis in his knee, and it worked incredibly well.

## Case presentation

**Figure 1:**
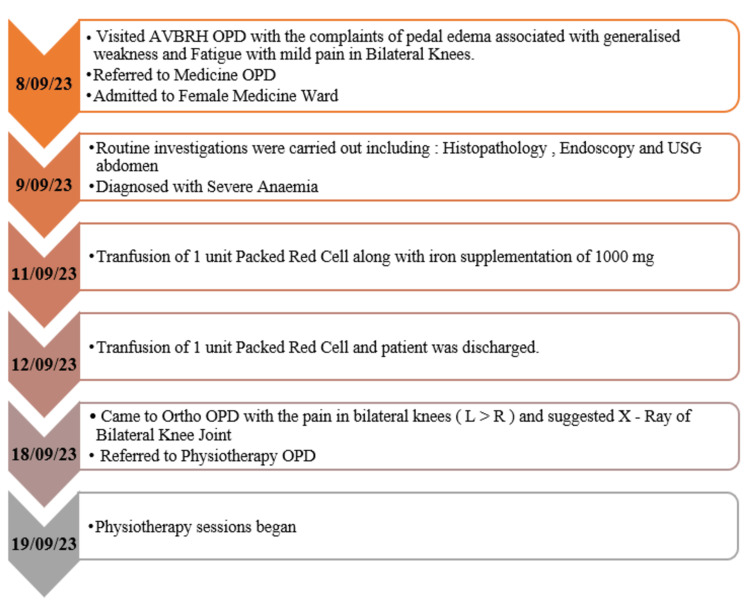
The timeline of the events. OPD: outpatient department; USG: ultrasonography; AVBRH: Acharya Vinoba Bhave Rural Hospital; L: left; R: right.

We are addressing the case of a 47-year-old female who was an employee at a general store. The patient appeared to be well one month prior to the development of bilateral pedal oedema on September 8, 2023. The oedema was of the pitting type and had neither an aggravating nor an alleviating factor. She also complained of slow-onset generalised weakness that got worse with activity and was better with rest. She also reported having mild pain in both of her knees. She visited Acharya Vinoba Bhave Rural Hospital (AVBRH) with the same concerns, and she was referred to the medicine department. After routine investigations like histopathology, endoscopy, and ultrasonography (USG) of the abdomen, she was diagnosed with anaemia and was admitted to the female medicine ward for eight days. She was discharged on September 12, 2023. Her bilateral knee pain worsened after a few days. She visited the orthopaedic department on September 18, 2023, for the same reason, and after an X-ray was taken, osteoarthritis in bilateral knees was diagnosed. She was then told to go to the physiotherapy outpatient department (OPD). The patient's current symptoms were stiffness in both knees in the morning and pain in both knees, particularly on the medial side of the knee joints (left > right). Long-term walking and the sit-to-stand transition caused more pain. Pain increased when sitting with folded legs and when flexing the knees. Figure 1 depicts the timeline of events.

Clinical findings

The physical examination was performed following the patient's informed consent. The patient was cooperative, awake, and aware of the person, place, and time. A thorough evaluation of the musculoskeletal system was conducted. There was no medical history for the patient. The examination of the bilateral lower limb, with particular attention to the knee joint, was carried out. Mild swelling was observed over the left knee joint as compared to the right. The pain was present over the medial joint line of the bilateral knee joint. Both of the knee joints had crepitus. The hip and ankle joints of bilateral lower limbs exhibited painless and complete quality of movement. The knee flexion for the left knee was 0 to 125 degrees, exhibiting a painful and incomplete quality of movement. The knee flexion for the right knee exhibited a painful and complete quality of movement. The knee extension of bilateral knee joints exhibited a painful and complete quality of movement. The ankle, knee, and hip joints all had a full range of motion. The manual muscle testing for hip, knee, and ankle joints is mentioned in the outcome measure section. Measurements of limb girth and length revealed a typical discrepancy of 1 cm. In Figure 1, the order of events is described and clarified.

Diagnostic assessment

**Figure 2:**
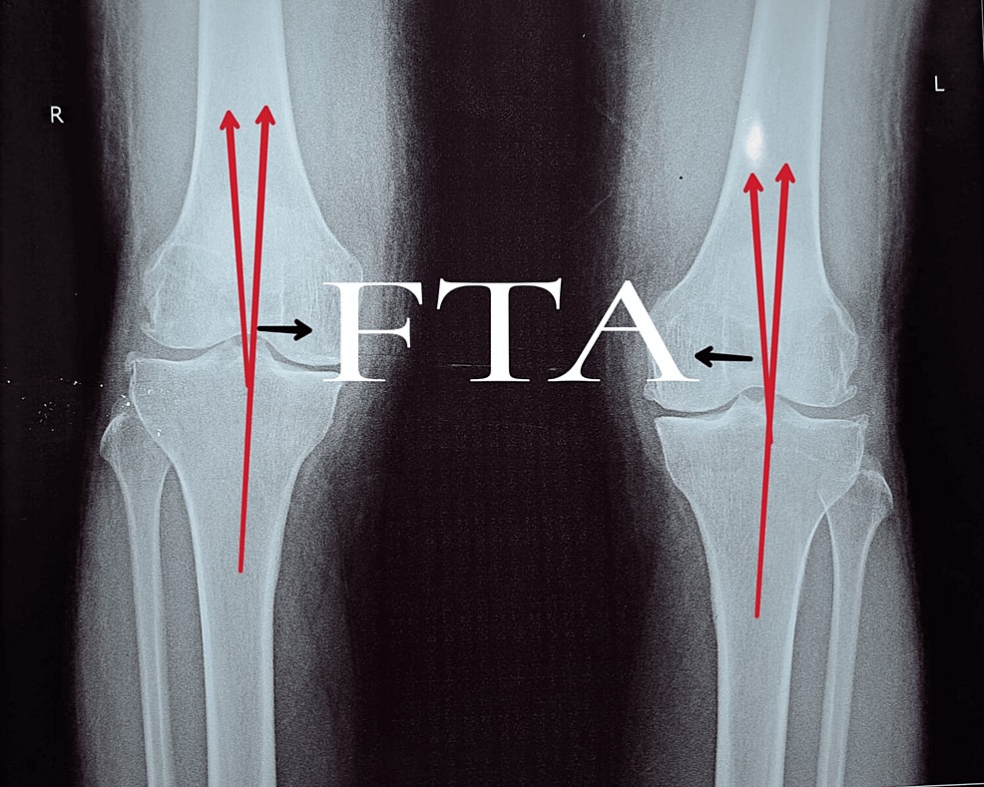
Medial joint space narrowing (left > right) in X-ray of bilateral knee joint in anteroposterior view. Femoral-tibial angle (FTA) for the right and left knee joints is depicted in the X-ray.

Eosinophilic duodenitis (eosinophilic index: 57/HPF) was suggested by the histopathology report and endoscopy, where a biopsy was obtained from D2. A USG of the abdomen showed evidence of splenomegaly. The bilateral knee joint's medial joint space was diminished (left > right) in an X-ray. Figure 2 shows the X-ray findings suggestive of grade 1 tibiofemoral and patellofemoral osteoarthritis. A femoral-tibial angle of six degrees is observed in the left and right knee joints.

Therapeutic intervention

Table 1 shows the goal-specific treatment based on the patient's difficulty with daily activities under the supervision of a physiotherapist.

**Table 1:** The treatment regimen for six weeks. Hz: Hertz; KT: Kinesio tape.

Goals	Intervention
To inform the patient about her health status, the importance of physical therapy rehabilitation, and the requirements that will be fulfilled for continued rehabilitation	Patient education involves informing the patient about osteoarthritis (OA), how to maintain an active lifestyle while protecting the joints, and how to control the disease's symptoms.
Pain relief and structural relaxation	Hot moist pack for 10 minutes [5]. Interferential therapy (IFT): Four interferential pad electrodes will be placed around the affected knee joint. Parameters: Frequency = 4000 Hz, base = 90 Hz, sweep = 40 Hz, beat frequency = 90-130 Hz, quadripolar duration = 10-20 minutes. IFT output intensity was increased until the “normal” tingling was encountered by the patient [6].
To enhance and develop muscular function	Exercises for quadriceps: quad sets (quadriceps setting), multiple-angle isometric exercises for quadriceps, short-arc terminal extension exercises for hamstrings: hamstring sets multiple-angle isometric exercises for hamstrings [7]. Three sets of 10 repetitions, each repetition with a five-second hold [5].
To improve range of motion and flexibility	Self-stretching technique taught for quadriceps and hamstrings. One set of three repetitions with a 30-second hold [5].
To alleviate pain and enhance joint mobility	Every day Mulligan mobilisation was used until the pain subsided. There were three sets of six repetitions given. Using a Mulligan belt, medial translational and medial rotational glides were delivered. The mobilisation was stopped as pain and movement improved [8].
To alleviate swelling and provide support to the joint	Based on the activation technique concept, the Kinesio tape was attached to the two quadriceps group muscles at a stretch of around 40% of its maximal length. Kinesio tape has been used on the two muscles of the quadriceps. (1) A Y-strip was placed to the inferior border of the patella for the rectus femoris (RF), and (2) a KT was attached to the vastus medialis (VM), 10 cm below the intertrochanteric line, to the medial border of the patella. Three applications were made on alternate days and three times a week [5].
To enhance muscle strength and functional activity	A resistance training programme was run. There was a provided eccentric training programme. For the hamstring and quadriceps muscles, we provided a variety of eccentric exercises. The training regimen was for two to four sets of eight to 12 repetitions, or 60% to 70% of one repetition maximum. Proprioception and functional activity were improved with the use of balance training. A range of activities was provided, including plyometric exercises, walking on their toes, retro walking, and leaning to the sides.

**Figure 3:**
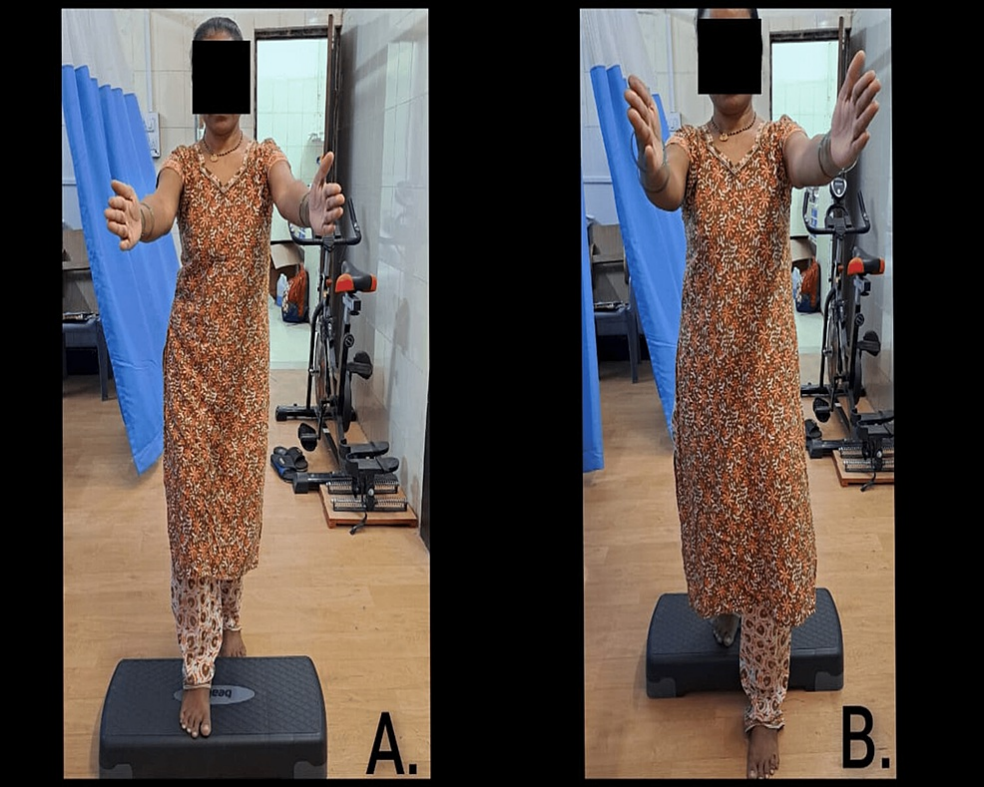
Balance training session. Forward step up with hand forward activity to enhance the regulation of balance.

**Figure 4:**
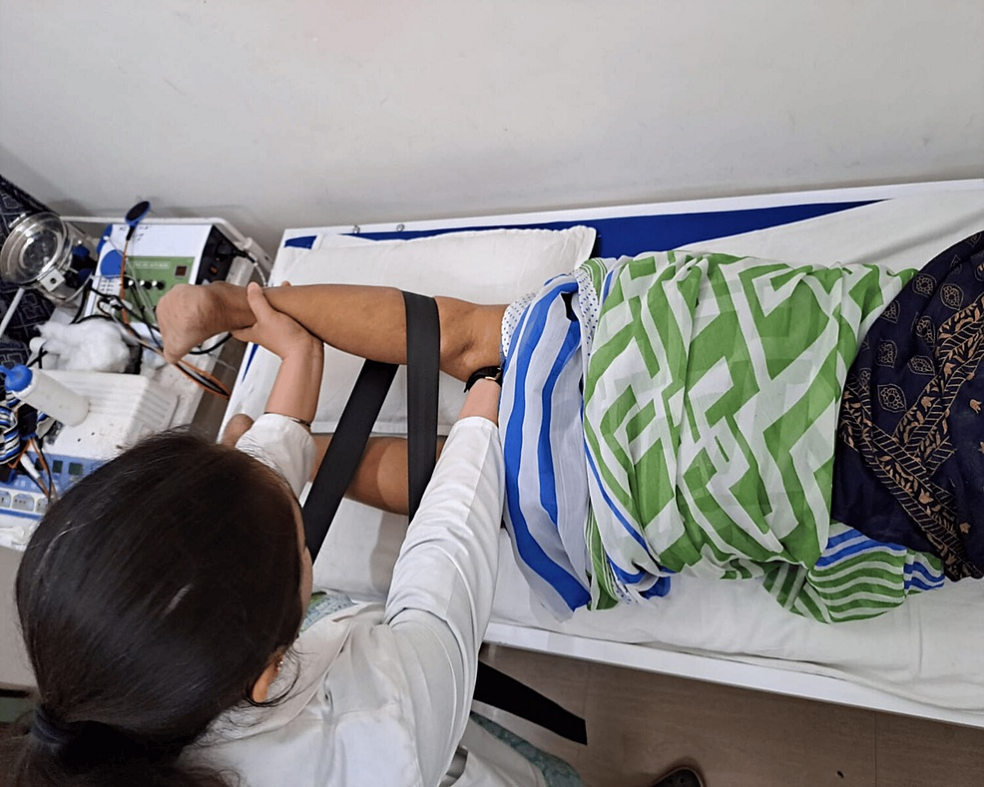
Mulligan mobilisation. Medial translational glide was administered using the Mulligan belt.

Figure 3 depicts the balance training exercises performed by the patient. Figure 4 depicts the Mulligan mobilisation administered using the Mulligan belt.

Follow-up and outcome of interventions

We utilized various outcome measures to analyse the results of our treatment regimen. Tables 2-4 depict the pre-rehabilitation and post-rehabilitation scores. The knee flexion of the left knee joint post-treatment was 0 to 135 degrees.

**Table 2:** Pre and post-treatment outcome measure scores. SF-36: 36-Item Short Form Survey; VAS: visual analogue scale.

Sr. No.	Outcome measures	Pre-treatment score	Post-treatment score
1.	Knee Injury and Osteoarthritis Outcome Score (KOOS)	48/100	69/100
	Pain	12	17
	Symptoms	7	10
	Activities of daily living	16	20
	Sports and recreation function	5	10
	Quality of life	8	12
2.	Western Ontario and McMaster Universities Osteoarthritis Index (WOMAC)	29/100	10/100
	Pain	6	3
	Stiffness	3	1
	Function	19	6
3.	SF-36 questionnaire	30/100	60/100
4.	VAS for right limb		
	On rest	1/10	0/10
	On activity	2/10	0/10
5.	VAS for left limb		
	On rest	2/10	0/10
	On activity	5/10	1/10

**Table 3:** Pre-treatment and post-treatment manual muscle testing grades of the right lower limb. Kendall's manual muscle testing grades were utilized. MMT: manual muscle testing.

Movements of the right lower limb	Pre-treatment MMT grades	Post-treatment MMT grades
Hip flexion	4+/5	5/5
Hip extension	4/5	5/5
Hip abduction	4/5	5/5
Hip adduction	4/5	5/5
Knee flexion	3/5	4/5
Knee extension	4/5	4/5
Ankle plantar flexion	5/5	5/5
Ankle dorsiflexion	5/5	5/5

**Table 4:** Pre-treatment and post-treatment manual muscle testing grades of the left lower limb. Kendall's manual muscle testing grades were utilized. MMT: manual muscle testing.

Movements of the left lower limb	Pre-treatment MMT grades	Post-treatment MMT grades
Hip flexion	4+/5	5/5
Hip extension	4/5	5/5
Hip abduction	3+/5	5/5
Hip adduction	4/5	5/5
Knee flexion	3/5	4+/5
Knee extension	3+/5	4-/5
Ankle plantar flexion	5/5	5/5
Ankle dorsiflexion	5/5	5/5

## Discussion

Osteoarthritis in the knee is a degenerative disease for which early detection is critical. Treatments can still be started during this critical period of the disease to stop it from progressing [9]. Numerous studies have demonstrated the value of conventional physical therapy regimens for individuals with osteoarthritis in the knee. Numerous modern physiotherapy methods, such as Mulligan and Kinesio taping, have demonstrated efficacy in treating this issue. Anam et al.'s study demonstrated that, for patients with osteoarthritis in their knees, Kinesio taping in conjunction with traditional physiotherapy has been proven to be far more beneficial than the muscle energy technique in conjunction with traditional physiotherapy [5]. Bhagat et al. demonstrated the efficacy of Mulligan's techniques in enhancing pain and functional mobility in patients suffering from osteoarthritis in the knee [8]. The improvement of this patient population is boosted when the traditional training programme is combined with these more contemporary strategies. Eccentric training is one type of programme that is highly helpful in enhancing lower limb strength and quality of life. Numerous studies on concentric and eccentric training, particularly in the context of knee osteoarthritis populations, have been conducted up to this point. Patients with knee osteoarthritis may potentially benefit more from eccentric training programmes [10]. Similarly, balance training is highly effective for improving proprioception and functional tasks [11]. We used this case study to implement a traditional treatment plan that included a number of contemporary physiotherapeutic techniques, such as Kinesio taping and Mulligan mobilisation, eccentric training programme, and balance training programme, for a patient with osteoarthritis in the knee. This case study demonstrates the efficacy of our treatment plan for osteoarthritis in the knee and suggests additional uses of it in clinical settings for this patient population.

## Conclusions

We created a treatment plan for a patient with osteoarthritis in the knee that included advanced physiotherapeutic techniques like the eccentric training programme, balance training programme, Kinesio taping, and Mulligan mobilisation. The patient's functional activities, quality of life, and pain were significantly improved after the treatment. Along with the balance training programme, the eccentric training programme significantly increased muscle strength and improved involvement in functional tasks. The patient's quality of life increased as a result. However, using recent physiotherapeutic techniques in addition to a conventional physiotherapy programme proved to be more advantageous in terms of enhancing the patient's general health and quality of life.
